# Whole genome sequencing data of the submerged macrophytes growth promoting and aerobic denitrifying bacterium Bacillus velezensis NBNZ-0060

**DOI:** 10.1016/j.dib.2023.109950

**Published:** 2023-12-12

**Authors:** Wenfeng Chen, Xinbo He, Yong Min, Jiaoli Zheng, Shimi Li, Yangfan Xu, Yaping Wang, Xiaoyan Liu, Yan Gong, Lei Zhu

**Affiliations:** a CCCC Second Harbor Engineering Company Ltd., Wuhan, Hubei Province 430040, People’s Republic of China; b National Biopesticide Engineering Technology Research Centre, Hubei Biopesticide Engineering Research Centre, Hubei Academy of Agricultural Sciences, Key Laboratory of Microbial Pesticides, Ministry of Agriculture and Rural Affairs, Wuhan, Hubei Province 430064, People's Republic of China; c State Key Laboratory of Biocatalysis and Enzyme, Engineering Hubei Collaborative Innovation Center for Green Transformation of Bio-Resources, Hubei Key Laboratory of Industrial Biotechnology, Biology Faculty of Hubei University, Hubei University, Wuhan, Hubei Province 430062, People's Republic of China

**Keywords:** Complete genome, Aerobic denitrifying bacterium, submerged macrophytes growth promotion, Bacillus velezensis, Lake sediment

## Abstract

The *Bacillus velezensis* strain NBNZ-0060 was isolated from the bottom sediment samples of the lake Jin in Wuhan, China. This strain is an aerobic denitrifying bacterium and able to promote growth of submerged macrophytes. The 3,929,784 bp entire genome contains 3,781 coding sequences (CDS), 27 rRNAs, 85 tRNAs, 5 ncRNAs, with an average G + C content of 46.5%. The average nucleotide identity and digital DNA-DNA values between strain NBNZ-0060 and *Bacillus velezensis* NRRL B-41580^T^ were 98.28% and 84.5%, respectively. The genome data have been deposited in NCBI with the accession number CP133277.1.

Specifications Table

**Table:** 

Subject	Biological sciences
Specific subject area	Microbiology, Genomics, Biotechnology
Data format	Raw, analyzed and assembled DNA sequences
Type of data	Table, Figure
Data collection	*Bacillus velezensis* NBNZ-0060 was isolated from the bottom sediment of the lake Jin in Wuhan, China. Pure culture of *B. velezensis* NBNZ-0060 was routinely cultured on lysogeny broth (LB) agar at 30°C. Genomic DNA samples were produced using modified SDS lysis method from exponential growth phase culture in LB medium at 30°C, 220 rpm, and purified by Omega column and Beckman AMPure XP Beads. The genome sequencing was performed on Oxford PromethION Nanopore sequencing platform and Illumina sequencing platform NovaSeq 6000.
Data source location	Sediment from China: Hubei, Wuhan, (114.1917°N, 30.6516°E)
Data accessibility	Data is publicly available at the NCBI GenBank from the following links: Genome data: https://www.ncbi.nlm.nih.gov/nuccore/2571260569 BioProject: https://www.ncbi.nlm.nih.gov/bioproject/1008384 BioSamples: https://www.ncbi.nlm.nih.gov/biosample/SAMN37118174 SRA Experiments: https://www.ncbi.nlm.nih.gov/sra/SRX21461174 https://www.ncbi.nlm.nih.gov/sra/SRX21461211

## 1 Value of the Data

• The genome data of Bacillus velezensis NBNZ-0060 can provide insights for the understanding of submerged macrophytes growth promotion potential.
• The experimental data and genome information may benefit researchers in the remediation of high nitrogen-contaminated sediments in lakes through the combined effect of microbes and submerged plants.
• The genome data could be useful for comparative genomic research of Bacillus strains with bioremediation capability.

## 2 Data Description

*Bacillus velezensis* NBNZ-0060 was obtained from sediment samples collected in the bottom of Jin Lake, China. The pot experiment of *Vallisneria natans* (Lour.) Hara growth in sediment enriched with nitrogen shows that strain NBNZ-0060 improved the survival rate by 225.0%, leaf length by 147.0%, root length by 132.0%, and fresh weight by 234.2%, respectively (Table 1). Furthermore, strain NBNZ-0060 exhibited a nitrate nitrogen removal capability of 75.8±2.7% after 24 hours of incubation at 100 mg/L nitrate under low oxygen conditions (cultivation without shaking).

**Table 1:** Effect of strain NBNZ-0060 on the survival number and biomass of *Vallisneria natans* (Lour.) Hara.

Treatments	survival number	survival rate (%)	Average leaf length (cm) per seedling	Average root length (cm) per seedling	Average fresh weight (g) per seedling
NBNZ-0060	18	60.0%	16.9 ± 1.8	3.3 ± 0.7	8.9 ± 2.1
CK	8	26.7%	11.5 ± 2.3	2.5 ± 1.0	3.8 ± 1.5

**Fig. 1:**
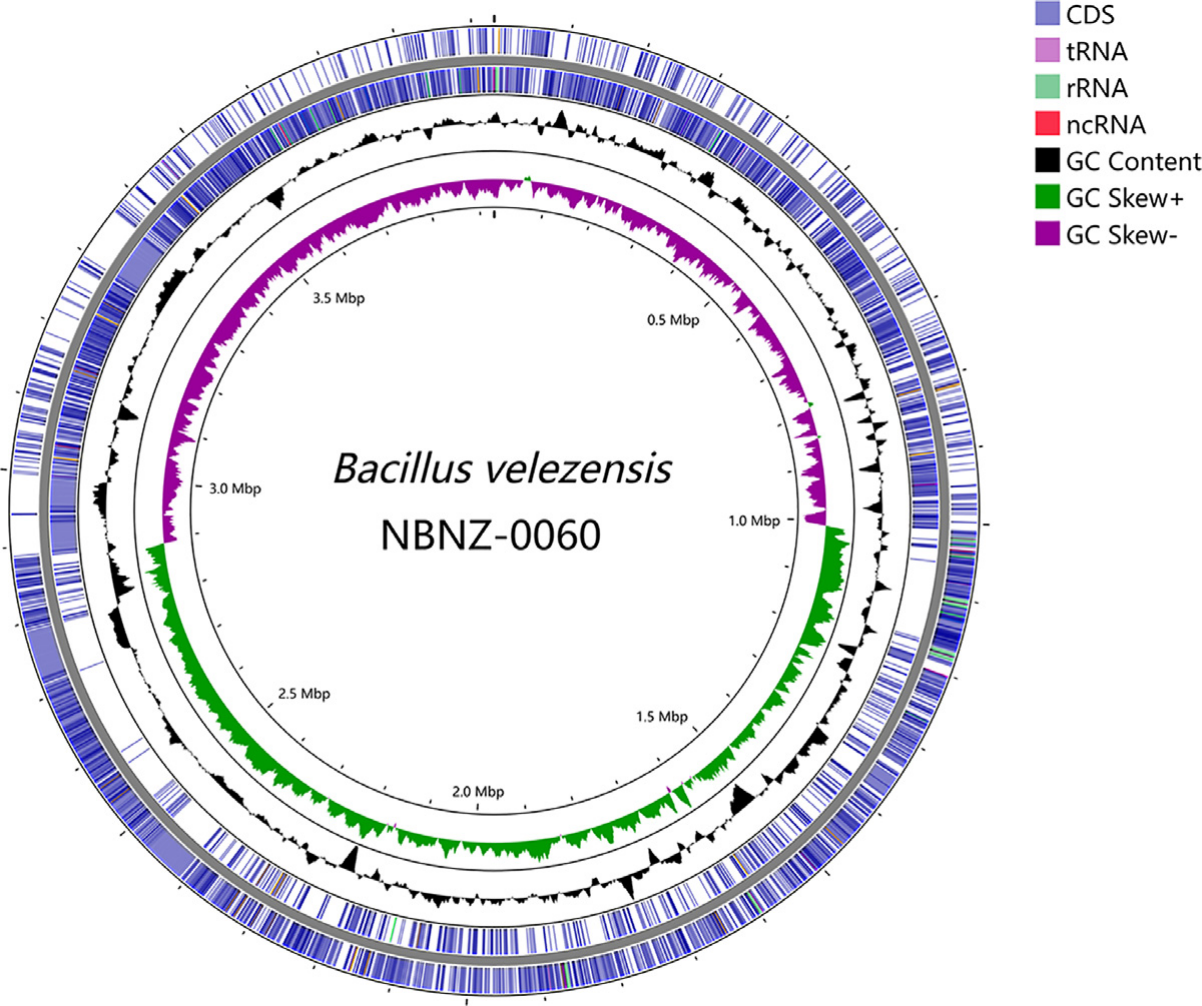
*Bacillus velezensis* NBNZ-0060 genome circular map with CDS (SlateBlue colour), tRNA (violet colour), rRNA (lime green colour), ncRNA (red colour), GC content (Black colour), Positive GC Skew (green colour) and Negative GC Skew (purple colour).

**Fig. 2:**
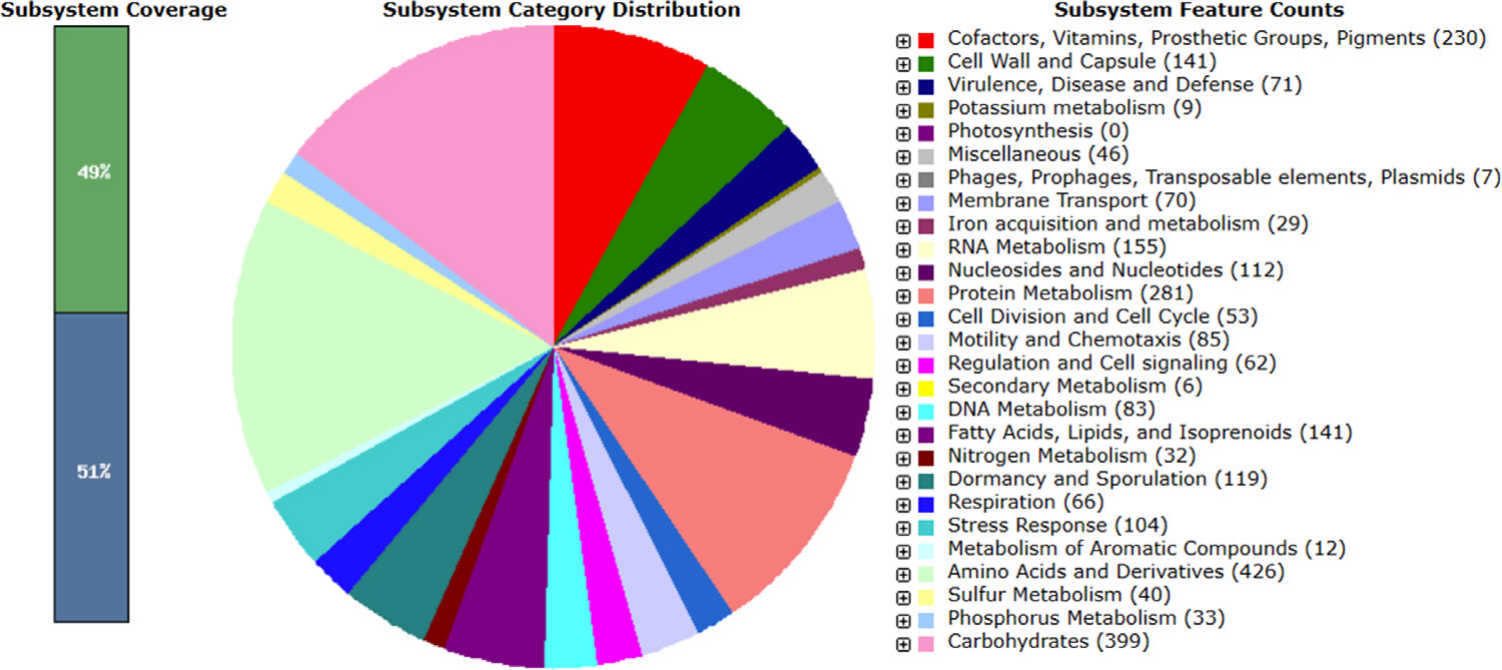
Subsystem category distribution of *Bacillus velezensis* NBNZ-0060 generated using the tool kit of the Rapid Annotation using Subsystem Technology (RASTtk). The bar chart shows the subsystem coverage in percentage (the blue bar corresponds to the percentage of proteins not identified). The pie chart shows the distribution of the 27 most abundant subsystem categories.

Table 2 summarizes genomic characteristics of *B. velezensis* NBNZ-0060. The chromosome of strain NBNZ-0060 is 3,929,784 bp in length, with a G+C content of 46.50%, and contains 3,781 CDS, 85 tRNA genes, 27 rRNA genes, as determined by NCBI Prokaryotic Genome Annotation Pipeline (PGAP) [2]. Fig. 1 shows a circular map of the NBNZ-0060 genome. The RAST server classified approximately 49% of the genome sequences into 26 of the 27 subsystems, yielding a total of 460 subsystems, as illustrated in Fig. 2. Based on the RAST annotation, the most abundant subsystem feature was metabolism of amino acids and derivatives (426 CDSs), followed by carbohydrates (399 CDSs), protein metabolism (281 CDSs) and vitamins metabolism (230 CDSs). 66 CDSs and 32 CDSs were assigned to the respiration and nitrogen metabolism respectively, which might be associated with denitrification in deep water. No discernible sequences were identified that could be applied to the photosynthesis subsystem.

**Table 2:** Genome features of *B. velezensis* NBNZ-0060.

Attribute	Value
Genome size (bp)	3,929,784
G + C content (%)	46.5
Subsystems	2,812
Genes (total)	3,898
Coding sequences (CDS, total)	3,781
Genes (coding)	3,680
Pseudo Genes	101
Genes (RNAs)	117
TRNAs	85
RRNAs	27
NcRNAs	5

Table 3 provides a concise overview of the potential genes associated with the indole-3-acetic acid (IAA) production in the NBNZ-0060 genome, with a focus on their role in promoting plant growth. The distribution of these genes primarily occurred within the indole-3-pyruvate pathway, tryptamine pathway, indole-3-acetonitrile pathway, and other related pathways [4]. In addition, a total of 12 biosynthetic gene clusters (BGCs) were identified, 7 of which matched known clusters with 86%∼100% similarity of bacillibactin, bacilysin, surfactin, macrolactin H, bacillaene, fengycin and difficidin, which exhibit varying degrees of antifungal and bacterial activity (Table 4) [6]. The remaining 5 BGCs with unknown products were predicted to encode saccharide (7% similarity), Class II lanthipeptides, type III polyketide synthase and terpenes (Table 4). The potential functions of these BGCs in the plant growth promotion could be further investigated.

**Table 3:** Putative genes involved in tryptophan dependent IAA production of *Bacillus velezensis* NBNZ-0060.

IAA synthesis pathways	Enzymes proposed in each step	Putative genes in NBNZ-0060 (locus tags and some annotated gene names)
indole-3-pyruvate pathway	amino transferase	RCF35_00290 (*patB*), RCF35_14490 (*bioA*), RCF35_15125
	indole-3-pyruvate decarboxylase	RCF35_07465, RCF35_12695 (*pyc*), RCF35_01945, RCF35_07080, RCF35_07085 (*bsdC*)
	indole-3-acetaldehyde dehydrogenase	RCF35_04555, RCF35_14870, RCF35_08890, RCF35_18825
tryptamine pathway	tryptophan decarboxylase	RCF35_07465, RCF35_12695 (*pyc*), RCF35_01945, RCF35_07080, RCF35_07085 (*bsdC*)
	indole-3-acetaldehyde dehydrogenase	RCF35_04555, RCF35_14870, RCF35_08890, RCF35_18825
indole-3-acetonitrile pathway	nitrilase	RCF35_09895
Others	tryptophan acetyltransferase	RCF35_03780

**Table 4:** Secondary metabolite clusters of *Bacillus velezensis* NBNZ-0060 as determined by antiSMASH.

Region	Genes	Type	The most similar known cluster	Similarity	Functions	Controlling Effects [6]
1	RCF35_00440-RCF35_00670	NRP-metallophore, NRPS, RiPP-like	bacillibactin	100%	Siderophore production	Microbial competitors
2	RCF35_03440-RCF35_03645	other	bacilysin	100%	Direct suppression	Bacteria, Cyanobacteria
3	RCF35_06930-RCF35_07135	NRPS	surfactin	86%	Biofilm, Induction of ISR	Fungi
4	RCF35_09955-RCF35_10155	PKS-like	butirosin A/butirosin B	7%	-	-
5	RCF35_10570-RCF35_10680	terpene	-	-	-	-
6	RCF35_11360-RCF35_11515	lanthipeptide-class-ii	-	-	-	-
7	RCF35_12415-RCF35_12640	transAT-PKS	macrolactin H	100%	Direct suppression	Bacteria
8	RCF35_13720-RCF35_13940	transAT-PKS, T3PKS, NRPS	bacillaene	100%	Direct suppression	Bacteria
9	RCF35_14340-RCF35_14655	NRPS, transAT-PKS, betalactone	fengycin	100%	Induction of ISR	Fungi
10	RCF35_14795-RCF35_14910	terpene	-	-	-	-
11	RCF35_15310-RCF35_15580	T3PKS	-	-	-	-
12	RCF35_16320-RCF35_16520	transAT-PKS	difficidin	100%	Direct suppression	Bacteria

The 16S rRNA gene sequence of strain NBNZ-0060 has been deposited to the NCBI GenBank database under accession number OR607840. *B. velezensis* NBNZ-0060 exhibited the highest similarity in its 16S rDNA gene sequence to *B. velezensis* CR-502^T^ (=NRRL B-41580^T^, 99.93%), *B. subtilis* NCIB 3610^T^ (99.79%), *B. siamensis* KCTC 13613^T^ (99.79%), followed by *B. nematocida* B-16^T^ (99.72%), *B. amyloliquefaciens* DSM 7^T^ (99.65%), *B. tequilensis* KCTC 13622^T^ (99.58%), *B. cabrialesii* TE3^T^ (99.58%), *B. inaquosorum* KCTC 13429^T^ (99.58%) and *B. stercoris* JCM 30051^T^ (99.58%) [7]. To determine the taxonomic affiliation of strain NBNZ-0060 at the genomic level, the average nucleotide identity (ANI) and digital DNA-DNA hybridization (dDDH) analysis indicated that NBNZ-0060 exhibits the highest ANI and dDDH values with *B. velezensis* KACC 13105 (99.97% and 99.9% respectively, Table 5).It is worth noting that *B. velezensis* KACC 13105 was initially classified as *B. methylotrophicus* but has since been reclassified as *B. velezensis* [10]. Furthermore, the ANI and dDDH values between NBNZ-0060 and *B. velezensis* NRRL B-41580^T^ were found to be 98.28% and 84.5% respectively. These values surpass the suggested threshold values for the species delineation (95–96% for ANI and 70% for DDH). This genome dataset places strain NBNZ-0060 in the species *Bacillus velezensis*.

**Table 5:** Taxonomic affiliation of *Bacillus velezensis* NBNZ-0060 based on ANI and dDDH values.

Reference strains	NCBI bioproject accession No.	Genome size (bp)	ANIb values	dDDH value
*Bacillus velezensis* KACC 13105	PRJNA267587	3,888,922	99.97%	99.9%
*Bacillus velezensis* FZB42	PRJNA13403	3,918,596	98.30%	85.3%
*Bacillus velezensis* NRRL B-41580^T^	PRJNA299292	4,034,335	98.28%	84.5%
*Bacillus siamensis* KCTC 13613^T^	PRJNA161489	3,779,696	94.43%	56.7%
*Bacillus amyloliquefaciens* DSM 7^T^	PRJEA41719	3,980,199	94.02%	55.4%
Bacillus subtilis NCIB 3610^T^	PRJNA29891	4,214,598	77.26%	21.0%

All strains with superscript capital T used for comparison were the type strains.

## 3 Experimental Design, Materials and Methods

### 3.1 Sample collection and bacterial isolation

Strain NBNZ-0060 was obtained from sediment samples. The samples were collected from the benthic region of Jin Lake (114.1917°N, 30.6516°E, Wuhan, China) during the month of May in the year 2021. A previously reported data involved the collection of a total of 25 sediment samples, each weighing between one and two kilos [1]. A dilution factor of 10 was applied to the sample by adding 1 g of the sample to 10 mL of sterile distilled water. Following this, the solutions with diluted gradients were uniformly spread onto lysogeny broth (LB) agar plates and incubated at 30°C for two days. The purification process involved subjecting individual colonies to repeated streaking on LB plates, yielding the isolation of strain NBNZ-0060 as a single pure colony.

### 3.2 Pot experiment of submerged macrophytes

The experiment was conducted using 30-cm high, 20-cm diameter plexiglass buckets with a 5-cm high layer of high nitrogen contaminated sediment (total nitrogen > 2000 mg/kg, collected from Jin Lake) and a 20-cm high layer of overlying water. Each bucket contained three seedlings of commercially available *Vallisneria natans* (Lour.) Hara, the initial height, and root length of which were cut and normalized to 5 cm, while the new weight of each treatment group remained relatively constant before planting. In each experimental group, ten seedlings were included. The containers were kept in a greenhouse at a constant temperature of 25°C and under light conditions of 14 h:10 h cycle. A volume of 1 mL of bacterial solution (1*10^8^ CFU/mL, grow in LB medium) was added to each container for the experimental group, while the control group received 1 mL of blank LB medium. The treatments was applied once a week for a total of three times. Following the completion of the entire 60-day experiment, number of surviving seedlings, fresh weight, leaf length, and root length of the seedlings in each group were measured and recorded.

### 3.3 Nitrate removal characteristics of strain NBNZ-0060

Denitrification medium (DM) were used for the nitrate removal experiment, containing 3.75 g glucose, 0.607 g NaNO_3_, 0.1 g MgSO_4_·7H_2_O, 3.00 g KH_2_PO_4_, 7.00 g K_2_HPO_4_, and 0.05 g FeSO_4_·7H_2_O per liter, pH 7.2. Following an overnight cultivation in LB medium, strain NBNZ-0060 was inoculated at 1% into DM medium with 100 mg/L nitrate and incubated at 30°C for 24 h without shaking. Then the culture were collected to measure the bacterial density (OD_600 nm_) and the concentrations of nitrate, using the supernatant fluid after centrifugation. A medium that did not contain incubated bacteria served as the blank control treatment and each experiment was replicated in triplicate. Nitrate was measured by phenol disulfonic acid photometry method.

### 3.4 Whole genome sequencing and assembly

Purified colonies of NBNZ-0060 were inoculated into LB medium and cultivated until reaching the mid-logarithmic growth phase, at 30°C, 220 rpm. Subsequently, genomic DNA isolation was performed using the Blood & Cell Culture DNA Kits (QIAGEN, Germany), following the guidelines provided by the manufacturer. The purity and concentration of DNA samples were assessed using the NanoPhotometer® spectrophotometer (IMPLEN, CA, USA) and Qubit® DNA Assay Kit in Qubit ® 2.0 Flurometer (Life Technologies, CA, USA). The monitoring of DNA degradation and contamination was conducted using 1% agarose gels.

For long-read sequencing, size-select of the genomic DNA sample were performed using the PippinHT system (Sage Science, USA). The DNA library was generated utilizing a ligation sequencing kit (SQK-LSK-109; Oxford Nanopore Technologies, Ltd. [ONT], Oxford, UK) without the application of DNA fragmentation. Subsequently, the library was subjected to sequencing using a Nanopore PromethION sequencer instrument (ONT) employing a R9.4.1 flow cell (FLO-MIN106). The process of base-calling and barcode segmentation, as well as the removal of adapter sequences from the raw sequences, was carried out using Guppy v.4.4.2 [11]. A total of 123,200 reads were produced, including 1,306,272,863 bp, which had an average length of 10,602.86 bp with a N50 value of 23,163 bp. For short-reads sequencing, the genomic DNA sample described above underwent fragmentation using sonication, utilizing the Covaris LE220 focused ultrasonicator (USA), yielding a size of 350bp. Subsequently, purification was carried out using AMPure XP magnetic beads (Beckman, Germany). A paired-end DNA library was created using the Truseq Nano DNA HT Sample preparation Kit (Illumina USA) in accordance with the guidelines provided by the manufacturer. The sequencing procedure was conducted on an Illumina NovaSeq 6000 equipment (Illumina, USA), using 150 nucleotide length reads. The raw data underwent processing using the FASTQ preprocessing software fastp v.0.23.2 [12] in order to remove adapters and low-quality data, yielding a total of 4,686,608 short reads, with a combined length of 1,405,982,400 bp. The assembly of the high-quality long- and short-read sequences was performed using Unicycler v.0.5.0 [13], yielding the generation of entire and circularized chromosomes. The average coverage is 361x.

### 3.5 Genome annotation and analysis

The complete genome was subjected to annotation using NCBI PGAP and RAST server [2,3]. The annotated genes were searched manually to identify the genes involved in IAA production. The prediction of BGCs were conducted using antiSMASH version 7.0.1 [5]. The 16S rRNA gene sequence was obtained using PCR amplification utilizing the universal primers 27F and 1492R [14], and compared using the EZBioCloud database [7]. The ANI calculator provided by CJ Bioscience's online platform was utilized to compute the ANI between genomes of strain NBNZ-0060 and its closely related species [8]. The dDDH analysis was performed using the Type (Strain) Genome Server (TYGS) [9].

## Limitations

Not applicable.

## Ethics Statement

This work does not involve human subjects or animal subjects. The authors declare that this manuscript is original work and has not been published elsewhere.

## CRediT authorship contribution statement

**Wenfeng Chen:** Conceptualization, Methodology, Software. **Xinbo He:** Data curation, Writing – original draft. **Yong Min:** Conceptualization, Resources. **Jiaoli Zheng:** Methodology, Formal analysis. **Shimi Li:** Methodology, Formal analysis. **Yangfan Xu:** Methodology, Formal analysis. **Yaping Wang:** Visualization, Investigation. **Xiaoyan Liu:** Writing – review & editing. **Yan Gong:** Validation, Supervision. **Lei Zhu:** Writing – review & editing, Supervision.

## Data Availability

SRA Experiments of Bacillus velezensis NBNZ-0060 (Original data) (NCBI SRA). BioSamples of Bacillus velezensis NBNZ-0060 (Original data) (NCBI BioSamples). SRA Experiments of Bacillus velezensis NBNZ-0060 (Original data) (NCBI SRA). BioProject of Bacillus velezensis NBNZ-0060 (Original data) (NCBI BioProject). Genome data of Bacillus velezensis NBNZ-0060 (Original data) (NCBI GenBank).
